# Antibiotic use practice and predictors of hospital outcome among patients with systemic bacterial infection: Identifying targets for antibiotic and health care resource stewardship

**DOI:** 10.1371/journal.pone.0212661

**Published:** 2019-02-22

**Authors:** Getachew Alemkere, Admasu Tenna, Ephrem Engidawork

**Affiliations:** 1 Department of Pharmacology and Clinical Pharmacy, School of Pharmacy, College of Health Science, Addis Ababa University, Addis Ababa, Ethiopia; 2 Department of Internal Medicine, School of Medicine, Addis Ababa University, Addis Ababa, Ethiopia; 3 Department of Pharmacology and Clinical Pharmacy, School of Pharmacy, College of Health Science, Addis Ababa University, Addis Ababa, Ethiopia; Universidade Catolica Portuguesa, PORTUGAL

## Abstract

**Background:**

Malpractice and excess use of antimicrobials have been associated with multiple costs, including the development of resistant bacteria, which has become a threat to the human health. The aim of this study, therefore, was to assess the antibiotic use practice and to identify predictors of hospital outcome to uncover targets for stewardship.

**Methods:**

An Institution-based prospective observational study was performed from 9 April to 7 July 2014 in the internal medicine wards of Tikur Anbessa Specialized Hospital. Patients with suspected systemic bacterial infections during this period were strictly followed and data were abstracted using data abstraction format. Descriptive statistics and binary logistic regression were used for statistical analysis.

**Results:**

About half of the attended patients had suspected systemic bacterial infections, in which pneumonia is the most common. Cephalosporins were the most widely prescribed class of drugs in all the wards. Initial antibiotics were empiric in almost all of the cases. About 28% of the ward and 59% of the ICU patients died during the in-hospital stay. The mean length of stay (LoS) was 18.5+12.2 in the wards and 8.9+4.9 days in the ICU. Whilst digestive disease (AOR = 6.94, 95% CI: 2.24, 21.49), different signs and symptoms of disease (AOR = 2.43, 95% CI: 1.30, 4.56), sepsis (AOR = 2.59, 95% CI: 1.12, 5.99) and vancomycin use (AOR = 2.60, 95% CI: 1.30, 5.21) were independent positive predictors, antibiotic days (> 10) (AOR = 0.37, 95% CI: 0.20, 0.70) was a negative predictor for mortality. On the other hand, hospital-acquired infection (AOR = 3.01, 95% CI: 1.05, 8.62), beyond the median antibiotic days (> 10) (AOR = 4.05, 95% CI: 1.96, 8.37) and agent days beyond 21 days (AOR = 2.18, 95% CI: 1.01–4.68) were independently associated with prolonged LoS.

**Conclusion:**

Generally, this observation entails an appropriate infection management and antimicrobial use policy. Any future policy should better start by addressing cases like pneumonia, and sepsis and drugs like cephalosporins.

## Introduction

About half of the antimicrobial agents prescribed to hospital in-patients are considered inappropriate [1]. This malpractice has been associated with multiple costs like the development of resistant bacteria [2,3]. As a result, it is more difficult than ever to challenge infections caused by antibiotic-resistant microbes [3].

The identification of infected patients at risk of poor hospital outcomes (e.g. in-hospital mortality) is important to provide an effective healthcare service [4,5]. Predicting hospital outcomes at admission and during the hospital stay may facilitate the healthcare delivery, as it can allow staff to manage healthcare resources optimally [2].

Different approaches have been promoted to save these precious drugs from the threat of resistant bacterial selection [6]. Antimicrobial stewardship is currently considered as the promising approach and has been promoted for all hospitals [5,7,8].

Although resistance is a global concern, it is primarily a local problem where single and multiple drug resistance to the commonly used antibiotics was high among bacterial isolates in different areas of Ethiopia [9,10], warranting rational use of drugs in the local environment. One study conducted in Tikur Anbessa Specialized Hospital (TASH) reported a high prevalence of multi-drug resistant bacterial strains that cause blood stream infection [11]. Thus, it needs a widespread effort at the individual institutional level to impact antimicrobial usage and, by extension (hopefully), antimicrobial resistance.

To the best of our knowledge, there was a dearth of studies done on the prudent use of antibiotics in TASH as well as in the country. However, other studies conducted in TASH reported suboptimal microbiologic reports utilization practice of healthcare professionals [12] and important gaps in their perception towards antimicrobial resistance [13]. The aim of the present study was therefore to perform a systematic and comprehensive assessment of antibiotic use practice and to identify predictors of hospital outcomes in hospitalized patients with systemic bacterial infections, in order to identify institutional targets for better antibiotic and health care resource stewardship. On the other hand, the aforementioned preliminary study on the perception of healthcare professionals in the hospital (conducted for a similar propose but after our study period) reported the need for specific educational priorities and implementation strategies [13]. Therefore, the study would have invaluable worth to supplement the hospital therapeutic decision, to the local health, for governmental decisions in the area, and for further studies.

## Materials and methods

### Study setting and period

TASH is a full-service 800-bed governmental University-affiliated tertiary care hospital in the country, Ethiopia. It provides ambulatory and in-hospital care. The in-hospital care is diversified, majorly involving the 120-bed internal medicine wards including a 6-bed medical intensive care unit (ICU). The study was conducted in all these internal medicine wards including the medical ICU since most adult cases were supposed to be seen in this unit. It was conducted from 9 April to 7 July 2014 for 3 consecutive months. Almost all the recording systems of the hospital during the study period were carried out manually. During the study period, the hospital had 4 infectious disease specialists and 2 microbiologists. An antimicrobial stewardship committee was fully established in November 2017 and currently active.

**Study Design:** The design was an institution based prospective observational study.

### Study population & sampling

All patients attending the adult internal medicine wards, including the medical ICU, of TASH during the study period and who had suspected systemic bacterial (non-mycobacterial) infections formed the study population.

**Fig 1:**
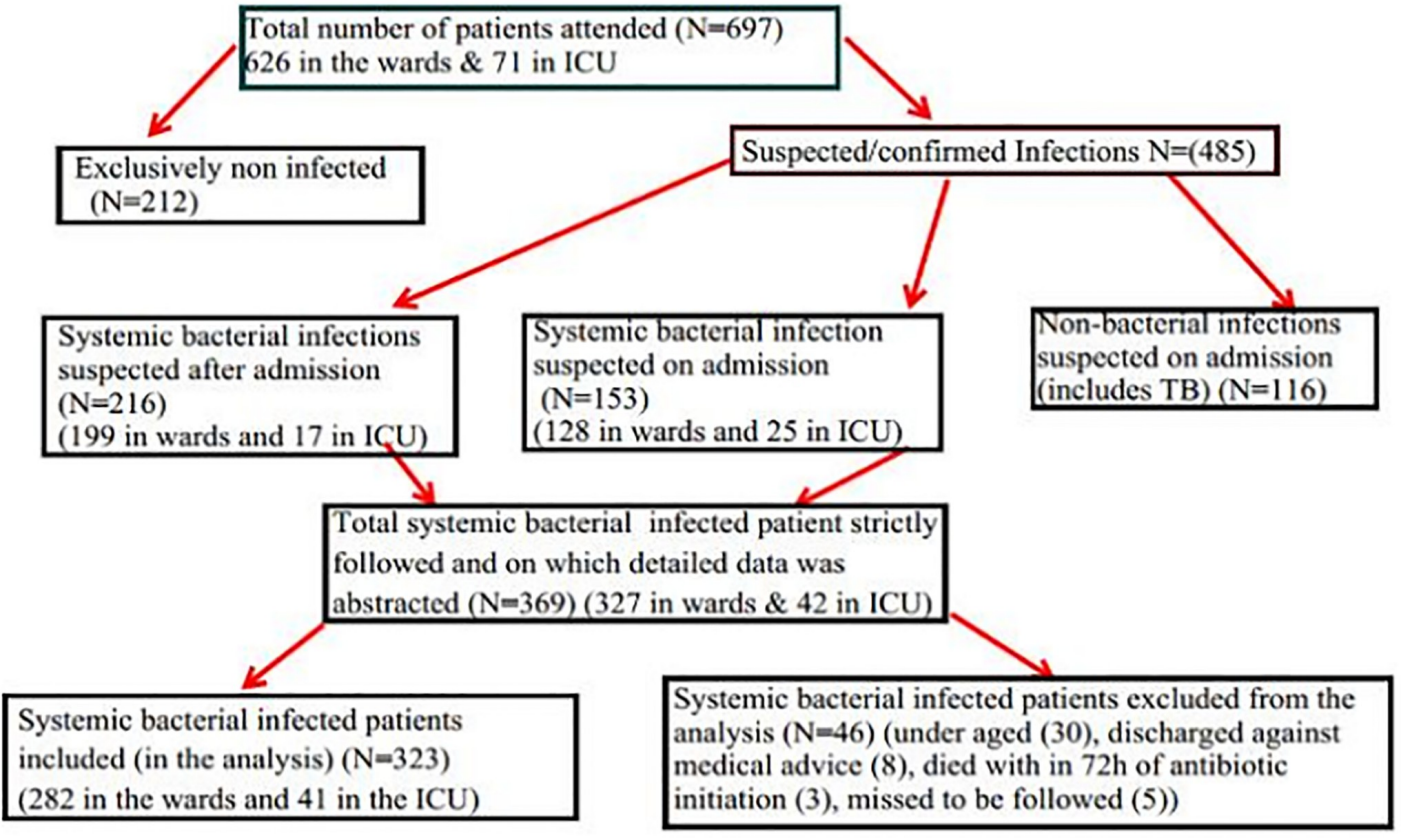
Patients included in the study among hospitalized patients with systemic bacterial infection in the internal medicine ward of Tikur Anbessa Specialized Hospital.

**Inclusion criteria**: Patients attending care in the adult internal medicine wards with suspected systemic bacterial infection and dispensed with systemic antibacterial agents during the study period were included. Patients taking antibacterial for < 72 hours, aged <18 years, lost to be followed up, or discharged against medical advice; patients taking anti-mycobacterial, non-systemic antibacterial, prophylactic antibacterial were excluded (Fig 1).

### Study variables

**Dependent Variables:** In-hospital mortality and prolonged length of stay (LoS) (taking the median value). **Independent Variables:** Socio-demographic factors like sex and age; Disease-related factors like primary/admission diagnosis, infection diagnosis, immunocompromised status, and glomerular filtration rates; Drug-related factors like different antibiotics, changes from initial therapy and antibiotic metrics; and Antibiotic use quality indicators for appropriateness of therapy.

### Data collection process

Data abstraction format was adopted from different literatures. The data collectors were four clinical pharmacy staff members of the hospital. To maximize the quality of the data, training, regular supervision and monitoring of the data collectors were performed. In addition, a pre-test was conducted on 5% of the initially assumed antibiotic user patients, outside of the study period. Patients who had (on admission) and developed (in the hospital stay) systemic bacterial (except mycobacterial) infections were strictly followed. The data collection for new admissions and abstraction of follow-up updates were performed on odd days bases by reviewing the patient sheet and consulting the attending healthcare provider(s). Demographic characteristics, admission diagnosis, suspected infection diagnosis, laboratory procedures performed (culture & gram staining) and the date of each laboratory report were documented properly. In addition, antibiotics administered, follow-up adjustments to the antibiotic regimen, and the dates’ of each antibiotic initiation and adjustment were also recorded properly. Criteria’s used for diagnosis, the microbiologic techniques, the decision to prescribe and modify antibiotics were left for the physician’s discretion. The data collectors deal with gaps evident during the data abstraction process with the health staffs, especially the attending physician. On biweekly basis, the principal investigator evaluates the work of the data collectors and gets unclear issues to the infectious diseases specialist.

### Data analysis

The collected data was checked, cleaned and double entered into epi info 7. Those records that did not satisfy the inclusion criteria and the intent of the data interpretation were excluded from the analysis, except for the rate of infection interpretation (Fig 1).

SPSS for windows version 21.0 was used for data analysis. Descriptive statistics and binary logistic regression were used for statistical analysis. Variables that exhibited a p-value of ≤0.05 in the univariable models were included in the multivariable logistic regression analysis in order to control confounding variables. Crude and Adjusted Odds Ratio (COR/ AOR) at 95% confidence level were calculated and finally the association was declared significant at *p<0*.*05*.

### Ethical consideration

Ethical approval was obtained from the Ethics committee of the School of Pharmacy, Addis Ababa University. In addition, the hospital management was requested for permission. Since the data collection was primarily dependent on patient charts, no written consent was requested from patients. However, information was given to patients, their physicians, and other health workers, as required. To ensure confidentiality, name and other identifiers of patients and prescribers were not recorded. The collected data was kept in a locked cabinet and only the researchers had access to it.

### Operational definitions

**Appropriateness of therapy:** It is based on the five quality indicators (File 1) developed after reviewing the patients’ records and the collected data, as proposed by van den Bosch *et al*. for adult non-ICU [14] and for sepsis [15]. These quality indicators were developed to fit our perspective based on the scientific requirements.

**Length of stay (LoS):** LoS was defined as the number of days (referenced by midnights) between admission and discharge, regardless of the number of hours, because the precise time was often not available. Prolonged LoS is a LoS above the median (> 16 days for the wards and > 10 days for the ICU).

**Signs and symptoms of the disease**: It is based on international classification 10 (ICD 10), which refers to the signs and symptoms of the underlying diseases (e.g. hemiparesis, secondary to hypertension) that were not classified elsewhere under the primary admission diagnosis but which had been the primary reasons for admission.

**Adjustment:** Changes made to the antibiotic/regimen after 48–72 hours of the initial therapy that refers to either of the following: **Discontinued**: meaning discontinuation of all antibiotics found to be unnecessary (e.g. no suspected infection); **Modified**: meaning either de-escalation (narrowing by either discontinuation of either agent or using the narrower spectrum option) or broadening (addition or using a much broader spectrum instead) of therapy.

**Antibiotic metrics**: Refers to the following antibiotic use measures: **Antibiotic courses**: a period during which the same systemic antibiotic (regardless of dose or route) was administered to the same patient on consecutive days; **Treatment periods**: a period of consecutive days on which any systemic antibiotic or combination of antibiotics was administered to a patient; **Agent days**: the number of days that a patient received a particular systemic antibiotic during the ward admission period; **Antibiotic days**: the number of days on which a patient received any systemic antibiotics during the ward admission period.

## Results

### Socio-demographic and disease characteristics of the patient

Patients had a mean age of 41.8± 17.8 (range: 18–85). Females accounted for about 52% of the study participants. Of all, 75.2% of the patients had suspected infection during ward admission. Patients with circulatory disease (34.4%) accounted for the second higher category of primary admission diagnosis (Table 1).

**Table 1:** Socio-demographic and disease characteristics of hospitalized patients with bacterial infection in the internal medicine ward of TASH in 2014, Addis Ababa, Ethiopia.

Variables	Wards, N = 282 (Freq, %)	ICU, N = 41 (Freq, %)	Total, N = 323 (Freq, %)
Average age (mean ± standard deviation (range))	41.7± 17.7 (18–85)	42.9 ± 18.9 (18–84)	41.8± 17.8 (18–85)
Sex of patient			
Female	149 (52.8)	20(48.8)	169(52.3)
Male	133(47.2)	21(51.2)	154(47.7)
Admission diagnosis (ICD 10)†			
Infectious diseases	209 (74.1)	34(82.9)	243(75.2)
Human Immunodeficiency Virus	45(16.0)	3(7.3)	48(14.9)
Circulatory disease	88(31.2)	23(56.1)	111(34.4)
Neoplasm	78(27.7)	2(4.9)	80(24.8)
Signs and symptoms of a disease	77(27.3)	8(19.5)	85(26.3)
Endocrine and metabolic disorders	33(11.7)	4(9.8)	37(11.5)
Digestive disorders	18(6.4)	3(7.3)	21(6.5)
Genitourinary disorders	23(8.2)	1(2.4)	24(7.4)
Blood-related disorders	22(7.8)	1(2.4)	23(7.1)
Respiratory disorders	20(7.1)	6(14.6)	26(8.1)
Other diagnosis††	19(6.7)	3(7.3)	22(6.8)
Abnormal organ functions			
Abnormal Renal Function Test	35(12.4)	11(26.8)	46 (14.2)
Glomerular filtration rate below 50 mL/min/1.73 m2	21(7.4)	6(14.6)	27 (8.4)
Microbiologic reports			
Gram stain reported	36 (12.8)	11(26.8)	47(14.6)
Culture reported	34(12.1)	4(10.81)	38 (14.9)
susceptibility was done*	9(81.8)	1(100)	10(83.3)
Origin of infection ♦			
Community-acquired	80 (28.4)	9(21.9)	89(27.6)
Hospital-acquired	35(12.4)	10(24.4)	45(13.9)
Unknown	167(59.2)	22(53.6)	189(58.5)
MDR risk ♦ ♦			
Absent	7(2.5)	2(4.9)	9(2.8)
Present	28(9.9)	12(29.3)	40(12.4)
Not enough evidence	247(87.6)	27(65.8)	274(84.8)
Immunocompromised ♦ ♦ ♦			
Yes	121(42.9)	7(17.1)	128(39.6)
No	161(57.1)	34(82.9)	195(60.4)

^†^a given patient may have >1 diagnosis, based on the International classification of disease (ICD);

^††^Other Diagnosis in wards: Drug adverse outcomes (8), Seizure/Epilepsy (4), gynecology (3), Arthritis (2), Communicable hydrocephalus (1) & Cholestatic calculi (1); ICU: Injury (2) Drug-related adverse outcomes (1));

*Denominator-all positive culture reports-12 for the total, 11 in the wards and 1 in the ICU;

^♦^ Origin of infection was classified based on the source of the infection labeled by the physician;

^♦ ♦^ Multi-drug resistance risk (MDR) criteria: prior antibiotic receipt in the past 3 months, previous hospital admission during the last 3 months, late-onset hospital-acquired infections (HAIs) (as defined by the physicians and/or the date of antibiotic administration relative to the admission date) (i.e. >5 days after admission), and presence of preexisting immunosuppressive disease;

^♦ ♦ ♦^ patients with febrile neutropenia, cirrhosis, disseminated TB & HIV infection were classified as immunosuppressed.

### Rate of infection

Using the total internal medicine ward admissions (697: 42 for ICU and 327 for wards) as the denominator, the systemic bacterial infection rate was 45.1% (282/626) for the wards and 57.7% (41/71) for the ICU. If the excluded patients with systemic bacterial infection were counted, the rate would have increased to 52.2% (327/626) for the wards and 59.1% (42/71) for the ICU (Fig 1). This rate, however, did not reflect the emergency department since the data collection was exclusively undertaken after the patients were admitted to the internal medicine wards.

### Infection diagnosis

Of all the patients, 48.0% had pneumonia. Community-acquired pneumonia in wards (25.2%) and aspiration pneumonia in ICU (36.6%) were the commonest types of pneumonia suspected (Table 2).

**Table 2:** Types of infections suspected in hospitalized patients in the internal medicine ward of TASH in 2014, Addis Ababa, Ethiopia.

Bacterial Diagnosis*	Wards, N = 282(Freq, %)	ICU,N = 41(Freq, %)	Total, N = 323(Freq, %)
Pneumonia	124(44.0)	31(75.6)	155(48.0)
Community-acquired	71(25.2)	7(17.1)	78(24.2)
Aspiration	27(9.6)	15(36.6)	42(13.0)
Hospital-acquired	21(7.5)	8(19.5)	29(9.0)
Ventilation associated	0	1(2.4)	1(0.3)
Other Pneumonia	5(1.8)	0	5(1.6)
Urinary Tract Infection	39(13.8)	3(7.3)	42(13.0)
Sepsis	34(12.1)	5(12.2)	39(12.1)
Fever of neutropenia	34(12.1)	1(2.4)	35(10.8)
Meningitis	18(6.4)	2(4.9)	20(6.2)
Abscess	15(5.3)	0	15(4,6)
Spon. Bacterial Peritonitis	11(3.9)	2(4.9)	13(4.0)
Gastroenteritis	11(3.9)	0	11(3.4)
Diabetic foot ulcer	10(3.5)	0	10(3.1)
Infective endocarditis	9(3.2)	0	9(2.8)
Skin infections	7(2.5)	1(2.4)	8(2.5)
Unknown infections	7(2.5)	0	7(2,2)
Parapneumonic effusion/empyema	6(2.1)	0	6(1.9)
Tetanus	0	2(4.9)	2(0.6)
Surgical site infections	0	1(2.4)	1(0.3)
Other Bacterial infections**	23(8.2)	1(2.4)	24(7.4)

*As per the labeling of the prescribing physician a given patient may have ≥ 1 bacterial diagnosis;

**Acute bronchitis (1), Acute Post Streptococcal Glomerulonephritis (1), Acute febrile illnesses (2), Chronic diarrhea (2), Cough (1), Emphysema (1), H. pylori (1), Intra-abdominal infections (4), lymphadenitis (Pyogenic) (2), Odontogenic infections (4), Osteomyelitis (2), Otitis Media (1), Pneumothorax (1), and Sore throat (1); for ICU: acute bronchitis; ICU: Intensive care unit

### Antibiotics and antibiotic related factors

#### Class of and specific antibiotics used

**Fig 2:**
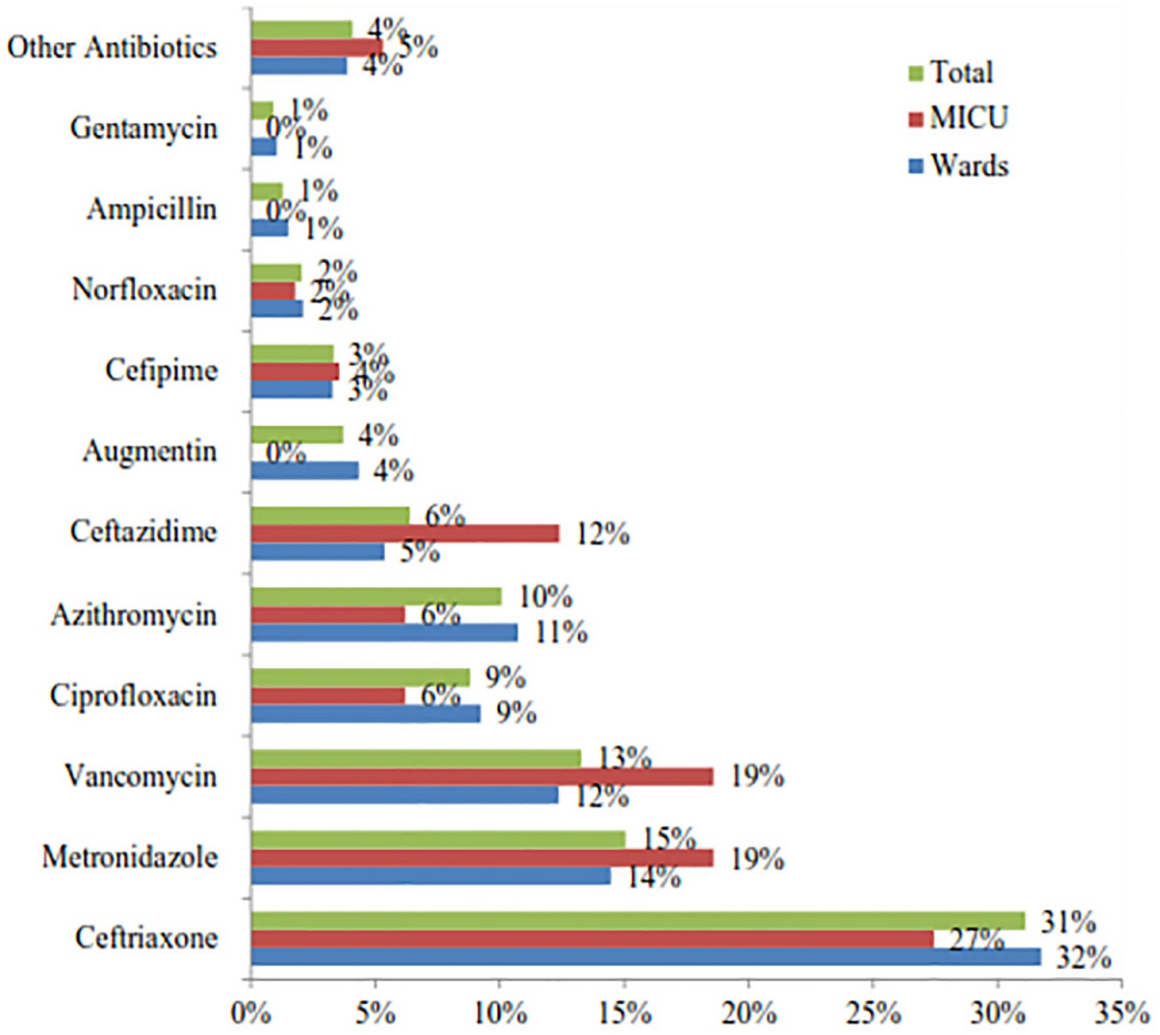
Types of antibiotics used in hospitalized patients with bacterial infection in the internal medicine ward of TASH in 2014, Addis Ababa, Ethiopia. Other Antibiotics: Wards: Clindamycin (5), Cloxacillin (4), Gemifloxacin (1), Cefotaxime (2), Cephalexin (1), Crystalline penicillin (1), Doxycycline (3), Meropenem (2), Chloramphenicol (3), Clarithromycin (2), Amoxicillin (2); ICU: Clindamycin (1), Clarithromycin (1), Amikacin (1), Doxycycline (1), Imipenem (1), Cotrimoxazole (1).

Comparatively (ward vs. ICU); cephalosporin (41% vs. 43%), anti-anaerobic (15% vs. 19%) and glycopeptides (vancomycin only) (12% vs. 19%) were the prevalently used class of drugs across the settings (Fig 2). The most frequently prescribed antibiotics were (ward vs. ICU) ceftriaxone (32% vs. 27%), metronidazole (14% vs. 19%) and vancomycin (12% vs. 19%). When grouped in a class comparatively (ward vs. ICU); cephalosporin (41% vs. 43%), anti-anaerobic (15% vs. 19%) and glycopeptides (vancomycin only) (12% vs. 19%) were the prevalently used class of drugs across the settings (Fig 2).

#### Antibiotic metrics

A given patient with a bacterial infection was exposed to 1–7 antibiotic courses with a mean of two or more. On average, a given patient with an infection had about two antibiotics simultaneously for both settings. The number of days that elapsed while the patient was on any antibiotic was 13.5 for the wards and 9.5 for the medical ICU (Table 3).

**Table 3:** Antibiotic use based on different metrics for hospitalized patients with bacterial infection in the internal medicine ward of TASH in 2014, Addis Ababa, Ethiopia.

Variable		Wards	ICU
Agent days, mean ± standard deviation (SD) (range (R))		23.2±19.5(2–135)	18.5±11.9(5–55)
Antibiotic days, mean ± SD (R)		13.5±19.6(2–51)	9.5±5.9 (3–30)
Antibiotics course, mean ± SD (R)		2.4 ±1.1 (1–7)	2.7±1.4 (1–7)
Maximum no. of antibiotics at a time, mean ± SD (R)		1.9±0.6 (1–4)	2.2±0.8(1–5)
Treatment periods	One	237(84.0%)	38(92.7%)
	Two	39(13.8%)	2 (4.9%)
	Three	6(2.1%)	1(2.4%)

#### Changes to initial therapy

**Fig 3:**
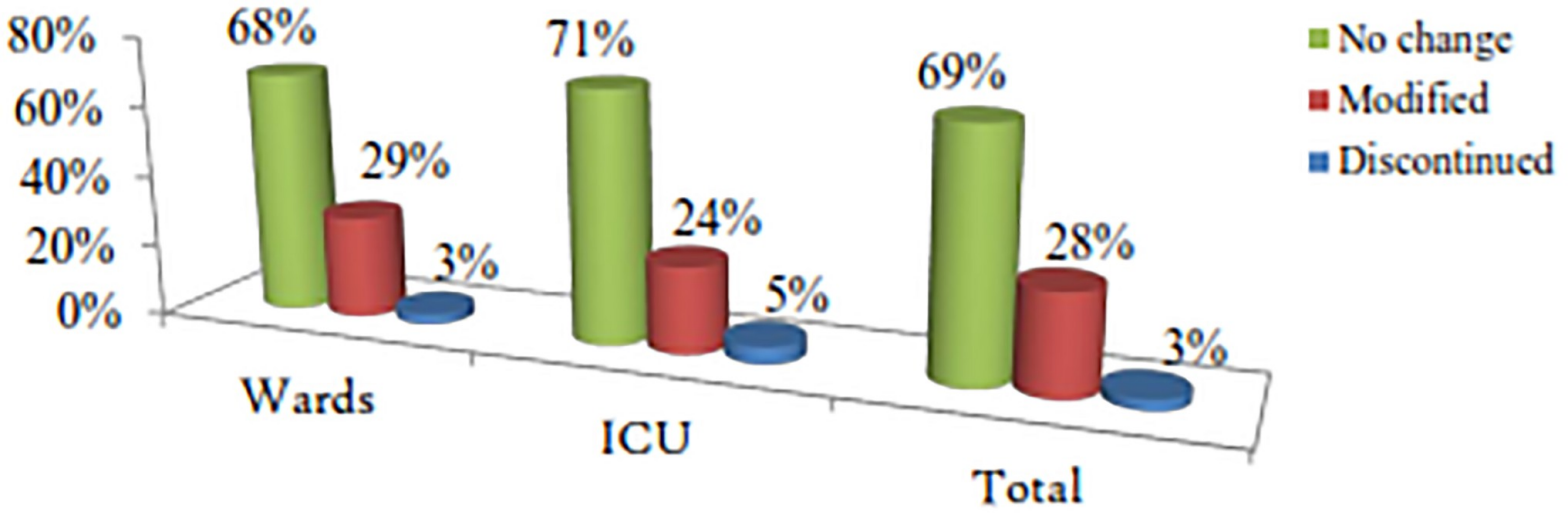
Adjustments to the initial antibiotic therapy for hospitalized patients with systemic bacterial infection in the internal medicine ward of TASH in 2014, Addis Ababa, Ethiopia.

This portion specifically deals with the initial therapy and its adjusted component. Adjustments not related to this were not addressed here. All patients in the ICU and almost all (99.6%) patients in the wards (except one (0.4%)) were started with empiric therapy. The initial therapy was adjusted only in a quarter of admitted patients (Fig 3).

### Appropriateness of antibiotic use

About 80% of the ward and 90% of the ICU patients had empiric antibiotics prescribed according to international guidelines. On the other hand, among the total 34 culture reports originated from the wards (Table 1), only 5 of them were available within 72 h of antibiotic initiation. Three of the available reports were negative and thus susceptibility was done for the two positive cultures only. The changes made for these 2 were based on the susceptibility report and thus taken as appropriate. However, no discontinuation of the empiric antibiotic therapy was performed for negative culture reports in all of the wards, thus considered to be inappropriate (Table 4). Although 238 wards admitted patients with an intravenous antibiotic survived to the required date, 20 were not candidates for oral therapy (see the Annex for limiting conditions). Among the 218 candidates, only 15 (6.9%) of them had oral switches (Table 4).

**Table 4:** Appropriateness of antibiotic use based on the five quality indicators of antibiotic use among hospitalized patients with systemic bacterial infection in the internal medicine ward of TASH in 2014, Addis Ababa, Ethiopia.

Quality indicators for appropriate antibiotic usage	Appropriate (Frequency(Percentage))	
	Wards	ICU
Empiric therapy is according to the guidelines	226 of 281 = 80.4%	37 of 41 = 90.2
Empiric therapy correctly changed according to culture susceptibility result reported within 72 h	2 of 2 susceptibility reports = 100%	-
Empiric therapy discontinued within 5 antibiotic days due to lack of culture reports	0 of 3 culture negative reports	-
Dose and dosing interval adapted to renal function	8 of 21 with GFR < 50 mL/min/1.73 m2 = 38.1%	1 of 6 = 16.7%
Intravenous to oral changes made within 5 antibiotic days	15 of 218 patients who had switches within≤5 antibiotic days = 6.9%	-

### Hospital outcome indicators

There was high mortality among patients with systemic bacterial infection in the hospital, 27.13% in the wards and 58.5% in the ICU. The LoS (in terms of mean ± standard deviations (range)) for wards and ICU was 18.5 ±12.2 (3–60) and 8.9±4.9 (3–23), respectively. ICU patients also spent 6.3±9.7 (2–41) days in non-ICU internal medicine wards, before or after their admission to the ICU (Table 5).

**Table 5:** Hospital outcome indicators in hospitalized patients with systemic bacterial infection in the internal medicine ward of TASH in 2014, Addis Ababa, Ethiopia.

Variables	Wards, n = 282 (Freq, %)	ICU, n = 41(Freq, %)	Total, n = 323 (Freq, %)
Final status of the patient			
Dead	78(27.7)	24(58.5)	102(31.6)
Discharged	204(72.3)	17(41.5)	221(68.4)
LoS (mean ± SD)	18.5±12.2(3–60)	8.9±4.9(3–23)	17.3±11.9(3–60)
LoS outside (for ICU only)	0	6.3±9.7(2–41)	-

### Predictors of hospital outcome

The predictors presented below were for the wards only.

#### Predictors of mortality

Digestive diseases (AOR = 6.94, 95% CI: (2.24, 21.49), p = 0.001) and different signs and symptoms of disease (AOR = 2.43, 95% CI: (1.30, 4.56), p = 0.005) of the primary admission diagnosis, sepsis (AOR = 2.59, 95% CI: (1.12, 5.99), p = 0.026) among infection diagnosis and vancomycin use (AOR = 2.60, 95% CI: (1.30, 5.21), p = 0.007) were independent positive predictors. Antibiotic days above ten (AOR = 0.37, 95% CI: 0.20, 0.70), p = 0.002) was a negative predictor (Table 6).

**Table 6:** Binary logistic regression analysis for predictors of mortality for hospitalized patients with systemic bacterial infection in the internal medicine ward of TASH in 2014, Addis Ababa, Ethiopia.

Variables	Mortality (yes) (%)	COR (95% CI)	AOR (95% CI)
Admissions diagnosis of signs & symptoms (yes)	29(37.7)	1.92 (1.20, 3.37)*	2.43 (1.30, 4.56)**
Admissions diagnosis of digestive problems (yes)	11(61.1)	4.62 (1.72, 12.40)**	6.94 (2.24, 21.49)**
Admission diagnosis of HIV (yes)	20(44.4)	2.47 (1.28, 4.77)**	1.53 (0.70, 3.37)
Sepsis (yes)	8(44.4)	3.53 (1.69, 7.34)***	2.59 (1.12, 5.99)*
Immunosuppressed (yes)	43(35.5)	1.98 (1.17, 3.37)**	1.46 (0.77, 2.77)
Vancomycin (yes)	32(39.5)	2.20 (1.27, 3.83)**	2.60 (1.30, 5.21)**
Antibiotic days (median) (> 10) (yes)	31(21.7)	1.85 (1.086, 3.138)*	0.37 (0.20, 0.70)**

*p < 0.05;

**p< 0.01;

***p<0.001;

HIV: human immunodeficiency virus; COR: crud odds ratio; AOR: Adjusted odds ratio; CI: confidence interval

#### Predictors of prolonged length of stay

The analysis revealed that presence of hospital acquired infection (AOR = 3.01, 95% CI: (1.05, 8.62) p = 0.040), antibiotic days beyond the median (>10 days) (AOR = 4.05, 95% CI: (1.96, 8.37), P = 0.000) and agent days beyond the median (>15 days) (AOR = 2.18, 95% CI: (1.01, 4.68), P = 0.046) were positively associated with prolonged LoS. Whereas presence of meningitis infection was negatively associated with prolonged LoS (AOR = 0.25, 95% CI: (0.07, 0.93), p = 0.039) (Table 7).

**Table 7:** Binary logistic regression analysis for predictors of prolonged length of stay for hospitalized patients with systemic bacterial infection in the internal medicine ward of TASH in 2014, Addis Ababa, Ethiopia.

Variables	Prolonged LoS (> 16 days) (yes) (%)	COR (95% CI)	AOR (95% CI)
Aspirational pneumonia (yes)	7(25.9)	0.38 (0.16, .93)*	0.56 (0.21, 1.52)
Hospital acquired pneumonia (yes)	17(81.0)	5.65 (1.85, 17.26)**	1.18 (0.28, 4.96)
Meningitis (yes)	4(22.2)	0.22 (0.06, 0.77)*	0.25 (0.07, 0.93)
Origin of infection			
Unknown	69(413)	1.00	(Reference)
Community Acquired	36(45.0)	1.16 (0.68, 1.99)	1.83 (0.93, 3.59)
Hospital acquired	24(68.6)	3.20 (1.42, 6.74)**	3.01 (1.05, 8.62)
Ceftazidime (yes)	27(75.0)	3.74 (1.64, 8.52)**	2.64 (0.90, 7.73)
Vancomycin (yes)	50(61.7)	2.00 (1.18, 3.41)*	0.80 (0.34, 1.89)
Ciprofloxacin (yes)	37(61.7)	2.16 (1.18, 3.91)*	1.28 (0.60, 2.75)
Antibiotic days (median) (> 10 days)	104 (72.7)	5.95 (3.56, 9.96) ***	4.05 (1.96, 8.37)
Agent days (median) (> 15 days)	19(30.2)	4.24 (2.58, 6.99)	2.18 (1.01, 4.68)
Adjustment to Empiric therapy			
No change	74(38.3)	1.00	(Reference)
Modified	54(66.7)	3.22 (1.86. 5.55)***	1.07 (0.51, 2.27)
Discontinued	1(12.5)	0.23 (0.03, 1.91)	1.99 (0.39, 10.12)

*p < 0.05;

**p< 0.01,

***p< 0.001;

COR: crud odds ratio; AOR: Adjusted odds ratio; CI: confidence interval

## Discussion

Consistent with a review report in low and middle-income countries [16], pneumonia was the most common infection in hospitalized patients. Unlike studies conducted in regional hospitals of Ethiopia where penicillins’ were the number one medications prescribed [17,18], cephalosporins were the most commonly used drugs in our setup. Comparable to previous studies in the hospital [19], empiric therapy was initiated for more than 99.6% of patients in the wards and all patients in the ICU of the present study. The studies conducted in TASH [19], including the current, were in complete disagreement with a study performed in one teaching hospital [20], where empiric therapy was initiated only in 19.4% of the patients.

One of the important issues in stewardship is the need assessment performed in line with hospital outcome indicators [4]. Being one of the outcome indicators, prolonged LoS was enormously associated with higher hospital costs [21]. The mean LoS reported for the medical wards in this study (18.5 days) was about four times higher than reported in Pakistan (4.74 days) [22] and six times of Iran report (3.02 days) [23] for the general patients, and as well as about 2 times higher than reported in Switzerland (9.8 days) [24] for pneumonia cases. The most probable reasons for an extended LoS observed in TASH might be related to the hospital system gaps like waiting for diagnostic or therapeutic procedures, or delay in discharge [23]. Another possible explanation could be due to the presence of MDR bacterial strains that could potentially extend the in-hospital daycare [25]. The present study also showed about a three-fold and four-fold higher ICU mortality than reported in high (20%) and low (15.4%) antibiotic resistance countries, respectively [26]. On the other hand, a study conducted among pneumococcal bacteremia patients in 21 hospitals in 10 countries (including developed and developing countries) reported a mortality rate of 16.9% [27]. This was lower than the ward mortality found in the current study (27.7%). Another study conducted in a Gambian hospital [28] reported an overall mortality of 6% and bacteremia attributed mortality of 8.3%, which is above 3 times lower than reported in this study. All these collectively indicate that the mortality in the current study was incomparably high, seeking an immediate attention.

Since the predictors in the medical ICU did not reach statistical significance, the points in the subsequent discussion were solely for the medical wards, unless otherwise indicated.

Among the primary admission diagnosis, in agreement with different studies [29,30] different signs and symptoms of diseases (AOR = 2.43, 95% CI: (1.30, 4.56), p = 0.005) like hypotension were associated with mortality. Digestive disorders (AOR = 6.94, 95% CI: (2.24, 21.49), p = 0.001) were also associated with mortality. Digestive disorders based on ICD-10 in our study encompass liver-cirrhosis, which might be the most probable reason for death in the current study [31]. Among the infection diagnoses, sepsis had a profound association with mortality both in the univariate (p<0.001) and multivariate (AOR = 2.59, 95% CI: (1.12, 5.99), p = 0.026) models. Accordingly, antimicrobial stewardship programs should make it a prime concern [16,32].

In the current study, patients using vancomycin were more than 2 times more likely to die (39.5%) both in the univariate and multivariate binary logistic models (COR = 2.20, 95% CI: 1.27–3.83, p<0.01; (AOR = 2.60, 95% CI: (1.30, 5.21), p = 0.007). Although no studies were found with a similar methodological approach in support of our evidence, this could possibly be explained by the inappropriate use of the drug in the hospital. Based on our assessment using a quality indicator investigational tool that included dosing for renal function (S1 File), vancomycin misuse had been the first most reason. In addition, one vancomycin use evaluation study conducted in the internal medicine ward of TASH revealed that vancomycin dose was not adjusted or adjusted inadequately in 96.5% of the cases [33]. Another possible explanation may be attributed to the different complications that are inherent in the drug’s pharmacology [34] and the emergence of resistant strains that potentially decrease the drug’s outcome [34,35]. In the current study, patients with prolonged antibiotic days (>10 days) were less likely to die AOR = 0.37, 95% CI: 0.20, 0.70), p = 0.002). Prolonged antibiotic exposure, in fact, is associated with multiple drawbacks like the emergence of antibiotic resistance [21,22]. Several studies attempted to address this concern and compared shorter (one-week) versus longer treatment durations and found no difference in outcome [36,37]. The difference observed between the current and those studies might be methodological, including setting a cut-off date for mortality. Hence, using cut-off date mortality, correlational timing relative to antibiotic initiation and death, and using other advanced methodological options could best reveal this association.

In contrast with an observational study in Italy [26], prolonged LoS did not have an association with any of the primary admission diagnoses. Similar to other studies [21,22], however, hospital-acquired infection (AOR = 3.01, 95% CI: (1.05, 8.62) p = 0.040) was associated with a prolonged hospital stay. In addition, prolonged antibiotic days beyond the median (>10 days) (AOR = 4.05, 95% CI: (1.96, 8.37), P = 0.000) and prolonged agent days (beyond the median >15 days) AOR = 2.18, 95% CI: (1.01, 4.68), P = 0.046) were independently associated with prolonged LoS. This may imply that patients will stay admitted until they finish their medication or the antimicrobial drug treatment will be prolonged until the patient get improved for any other clinical scenarios.

Given the cost of combination therapy, guidelines restrict such treatment approaches for certain group of patients [38] and recommend prompt de-escalation based on the patient’s clinical course, and culture & susceptibility test results [36,37]. Despite this concept and Mettler *et al* [20] report, though almost all our patients started with broad-spectrum combination empiric therapies, the modification was done only for the quarter of the patients (29% for wards vs. 24% for ICU). Even these modifications did not necessarily indicate streamlining (lowering the estimate) since the majority of the modifications involved the addition of therapy for clinical deterioration, identification of new site of infection, and for culture-positive microbiologic reports [12,38].

Among the 5 quality indicators, only concordance to the guideline and intravenous to oral switch were tested for statistical association and found to be associated neither in the univariate nor in the multivariate model with both outcome indicators. The remaining 3 quality indicators had too low observations to test, with profoundly different denominators.

Since this study was a 3 month long prospective observational, unlike to the previous works, it has made a timely, relevant and comprehensive contribution in uncovering the facts for the prudent use of antibiotics. Being an observational analysis, however, it had limitations. The study was conducted in only one hospital, and practice patterns, patients’ characteristics and microbiology resistance patterns may vary among hospitals, which may limit its generalizability. The use of international guidelines might also under or overestimate the report. Because of the initial different design, there were some partially addressed factors (like the origin of infection and MDR risk status) and the recommendations based upon these may possibly introduce some bias. Although the use of multivariate analysis helped to control a substantial proportion of confounding variables, data related to some important variables like the severity of the illness, the presence of medical devices, previous ICU admission and antibiotic exposure status were not addressed. Therefore, all these might have affected the outcomes.

## Conclusion

This observation showed that about half of admitted patients had suspected infection and received antibiotics on an empiric basis. Almost none of the empiric antibiotics were justified based on microbiologic cultures. Whilst pulmonary infections were the most frequent type of infections, cephalosporins were the most commonly prescribed drug class. Presence of digestive disease, different signs, and symptoms of the disease, sepsis, and vancomycin use were positive predictors of mortality. On the other hand, hospital-acquired infection, beyond the median antibiotic days (> 10 days) and agent days (>15 days) were independently associated with prolonged LoS. This suggests that local guidelines or any stewardship activities should give priority to all these issues. Future researchers in the hospital should better address ICU cases separately, focus on modifiable risk factors, use a time-to-event analysis and other advanced methodological designs.

## Supporting information

S1 File The five quality indicators used to measure the appropriateness of antibiotic use among hospitalized patients with systemic bacterial infection in the internal medicine ward of TASH in 2014, Addis Ababa, Ethiopia. (DOCX)
