# Supplementary material for: Antibiotic use practice and predictors of hospital outcome among patients with systemic bacterial infection: Identifying targets for antibiotic and health care resource stewardship
Source: PLoS One. 2019 Feb 22;14(2):e0212661. doi: 10.1371/journal.pone.0212661 (PMC6386277; doi:10.1371/journal.pone.0212661)
Supplement: S1 File — (DOCX) [file pone.0212661.s001.docx]

## File 1: The five quality indicators used to measure the appropriateness of antibiotic use among hospitalized patients with systemic bacterial infection in the internal medicine ward of TASH in 2014, Addis Ababa, Ethiopia

| s.no. | **Quality indicator**  All patients are: adults admitted to ICU and non-ICU internal medicine wards with > 72 h of systemic antibiotics because of a suspected systemic bacterial infection | **Numerator description**  All patients are: adults admitted to ICU and non-ICU internal medicine wards with > 72 h of systemic antibiotics because of a suspected systemic bacterial infection | **Denominator description**  All patients are: adults admitted to ICU and non-ICU internal medicine wards with > 72 h of systemic antibiotics because of a suspected systemic bacterial infection |
| --- | --- | --- | --- |
|  | Empirical systemic antibiotic therapy should be prescribed according to widely used guidelines, IDSA based (since no local guidelines) | Number of patients who started with empirical systemic antibiotic  therapy according to the guideline | Total number of patients who started with empiric systemic antibiotic therapy |
|  | Empiric antibiotic therapy should be changed to pathogen-directed as soon as culture results become available  **Culture reports for the adjustment were expected to be available within 48-72 hours of empiric therapy initiation, otherwise not considered* | No of patients with empiric systemic antibiotics whose culture reported positive within 48-72 hours and changing to pathogen-directed therapy was done appropriately  * *Appropriate is when the infective organism is also sensitive for a narrow spectrum antibiotic changed* | Total no of patients with empiric systemic antibiotics whose culture reported positive within 48-72 hours *and in whom changing to pathogen-directed therapy was done.* |
|  | Dose and dosing interval of systemic antibiotic should be adapted to renal function | No of patients with a compromised renal function who started with an empirical systemic dose adjusted to renal function. | Total number of patients who started with empirical systemic antibiotic therapy and who had a compromised renal function,  **defined as an estimated Glomerular filtration rate (GFR) < 50 ml/min/1,73m* |
|  | Empiric antibiotic therapy for presumed bacterial infection should be discontinued within 3-5 days if culture was reported to be negative | No of patients whose empiric antibiotic therapy is discontinued within 3-5 days, because of lack of microbiological evidence of infection. | A total number of patients who started empirical systemic antibiotic therapy, but lacked microbiological evidence of infection. |
|  | Systemic antibiotic therapy should be switched from intravenous to oral antibiotic therapy within 3-5 days of antibiotic initiation for all patients surviving to this date and continued with the treatment unless there is limiting conditions,  **Limiting conditions, When oral intake and gastrointestinal absorption are inadequate, (exceptions like endocarditis, meningitis)* | Number of patients with  intravenous antibiotics for 5 and more days, in whom changes to an oral agent was done. | Total number of patients  with intravenous antibiotics  for 5 and more days, in whom  changing to oral antibiotic  therapy was expected. |
